# Perceptions of health providers towards the use of standardised trauma form in managing trauma patients: a qualitative study from Tanzania

**DOI:** 10.1186/s40621-020-00244-3

**Published:** 2020-05-01

**Authors:** Hendry R. Sawe, Nathanael Sirili, Ellen Weber, Timothy J. Coats, Teri A. Reynolds, Lee A. Wallis

**Affiliations:** 1 Department of Emergency Medicine, Muhimbili University of Health and Allied Sciences, P.O. Box 65001, Dar es Salaam, Tanzania; 2 Division of Emergency Medicine, Faculty of Health Sciences, University of Cape Town, Cape Town, South Africa; 3 Department of Development Studies, School of Public Health and Social Sciences, Muhimbili University of Health and Allied Sciences, Dar es Salaam, Tanzania; 4 Emergency Department, University of California, San Francisco, CA USA; 5 Department of Cardiovascular Sciences, University of Leicester, Leicester, UK; 6 Unit Head, Clinical Services and Systems, Integrated Health Services, World Health Organization (WHO), Geneva, Switzerland; 7 Division of Emergency Medicine, Faculty of Health Sciences, University of Cape Town, Cape Town, South Africa

**Keywords:** Trauma registry, Standardized documentation, Provider perception, Africa, Tanzania

## Abstract

**Background:**

Trauma registries (TRs) are essential to informing the quality of trauma care within health systems. Lack of standardised trauma documentation is a major cause of inconsistent and poor availability of trauma data in most low- and middle-income countries (LMICs), hindering the development of TRs in these regions. We explored health providers’ perceptions on the use of a standardised trauma form to record trauma patient information in Tanzania.

**Methods:**

An exploratory qualitative research using a semi-structured interview guide was carried out to purposefully selected key informants comprising of healthcare providers working in Emergency Units and surgical disciplines in five regional hospitals in Tanzania. Data were analysed using a thematic analysis approach to identify key themes surrounding potential implementation of the standardised trauma form.

**Results:**

Thirty-three healthcare providers participated, the majority of whom had no experience in the use of standardised charting. Only five respondents had prior experience with trauma forms. Responses fell into three themes: perspectives on the concept of a standardised trauma form, potential benefits of a trauma form, and concerns regarding successful and sustainable implementation.

**Conclusion:**

Findings of this study revealed wide healthcare provider acceptance of moving towards standardised clinical documentation for trauma patients. Successful implementation likely depends on the perceived benefits of using a trauma form as a tool to guide clinical management, standardise care and standardise data reporting; however, it will be important moving forward to factor concerns brought up in this study. Potential barriers to successful and sustainable implementation of the form, including the need for training and tailoring of form to match existing resources and knowledge of providers, must be considered.

## Background

Trauma registries (TRs) are systems that provide timely injury data collection to support evaluation and monitoring of quality of care, and development of resource-specific treatment and prevention interventions (Mehmood et al., 2013; Nwomeh et al., 2006; Schultz et al., 2007). Despite burdens of trauma being highest in low and middle-income countries (LMICs), TRs are effectively non-existent in these regions (Haagsma et al., 2015; World Health Organization, 2008). In most high-income countries (HIC), the existence of formal trauma care systems incorporating functional TRs have significantly contributed to the reduction of injury morbidity and mortality (Cameron et al., 2004; Paradis et al., 2018). In these countries, the development and implementation of TRs required significant financial investment, human resource engagement and commitment to ensure high quality data can be collected and aggregated sustainably (Moore & Clark, 2008).

The use of TRs to document and interpret data on trauma and injuries is key to prioritising prevention efforts, monitoring injury diagnosis, management and outcomes in any trauma care system (Krug et al., 2000; Nordberg, 2000). However, in Africa, absence of formal trauma care systems and lack of TRs to generate accurate injury data make it difficult to clearly delineate the incidence, mechanism, and management of injury; this hinders the development of context-appropriate interventions to support the prevention of injuries and improve the quality of trauma care (London et al., 2001; Michaud & Murray, 1994; Smith & Barss, 1991).

In Africa, the implementation of TRs has been largely unsuccessful or unsustainable, with most registries being limited to single health facilities during a defined research period, with no sustainability past the research phase (Chalya et al., 2012; Kobusingye et al., 2002; Kobusingye & Lett, 2000). Despite this, developing and implementing standardized trauma form to support care process and evidenced based interventions remains a priority (Reynolds et al., 2014). Lack of a defined standardised injury set and absence of resources to centrally compile and analyse injury data are among most African countries’ documented reasons for failure to establish TRs (Nwomeh et al., 2006). In an efforts to standardise reporting and generation of trauma data, the World Health Organization (WHO) recently developed a standard data set for injury (DSI) through the WHO International Registry for Trauma and Emergency Care platform (WHO Dataset for Injury, n.d.). The WHO platform is available locally to each member country as to use as a national trauma registry, and the standardisation of data collected will allow for international comparability.

In order to address the lack of standardised injury data and prepare for a national TR, a standardised trauma form for trauma patients was developed using the WHO DSI (Sawe et al., 2020). This form was intended to facilitate both clinical care and data collection within healthcare facilities across Tanzania. This study aimed to describe healthcare providers’ perceptions on using standardised trauma form to manage trauma patients and collect key registry data at regional-level hospitals in Tanzania.

## Methods

### Study design

This exploratory qualitative study utilised key informant interviews with healthcare providers across the five hospitals. This study was conducted at five regional hospitals in Tanzania - Mwananyamala, Tumbi, Arusha, Morogoro and Tanga - between August 2018 and December 2018. The United Republic of Tanzania is a low-income country with a population of 55 million. It has a pyramidal health system structure spanning dispensaries, health centres, and district, regional and consultant hospitals (Tanzania, 2017). At the time of this study, Tanzania had a total of 25 regional hospitals. Five regional hospitals (representing 20% of all regional hospitals) were purposefully selected based on their representativeness of emergency care provision in the country. None of the included hospitals had formal documentation system for trauma cases; however, each hospital was mandated to document basic patient data in a Health Management Information System (HMIS) register book provided by the Ministry of Health (MoH). The HMIS records a small amount of data -only basic demographic information (age and sex), diagnosis, investigation, and disposition - and is intended to be submitted monthly to MoH (Ministry of Health, 2017).

### Sample population

Principal investigator chose the participants from each hospitals based on their involvement with care process of trauma patient. A purposive sampling strategy was employed in all five sites to ensure a maximum variation of cadres and work experience of the study participants. This included the Clinicians (Specialist Doctors, Medical Officers, Assistant Medical Officer, Clinical Officers), Nurses, Administrative staff (Hospital administrators, Information and Communication Technology officer, and Health Information Records Officers). The initial target sample size of 35 participants (7 from each site) was earmarked based on the proportion of staff that have role in the care process of trauma patients in each of these hospitals. However, as interviewes were being conducted, we realized that at the 33rd respondents there was no new relevant information coming out as we had attained information saturation and thus stopped data collection (Table 1). At this point even with different probing styles no new information, concepts or themes were disclosed, after which no additional interviews were conducted (Marshall, 1996) The sample size for the interview portion of the study was determined by the principle of theoretical saturation at each site.

**Table 1:** Hospital Roles of Study Participants

Hospital Role	Interview, ***N*** = 33 ***n*** (%)	Gender: male %	Prior use of trauma form %
Nurse	6 (18.2)	83.3	16.7
Medical Officer	8 (24.2)	75	37.5
Assistant Medical Officer	5 (15.2)	80	0
Clinical Officer	6 (18.2)	83.3	0
Specialist Doctor			0
Emergency Physician	1 (3)	100	100
Orthopaedic/Trauma Specialist	1 (3)	100	0
Surgery Specialist	1 (3)	100	0
Administrator	2 (6.1)	50	0
HMIS Officer	2 (2.1)	50	0
ICT Officer	1 (3.0)	100	0

### Data collection

Interviews were conducted by the principal investigator (specialists emergency physician) with short course training in qualitative research methodology. A semi-structured interview guide, with questions aimed at exploring the perceptions surrounding the use of standardised clinical charts versus current trauma patient data documentation practices (Dicicco-Bloom & Crabtree, 2006; Mays & Pope, 2000). Interviews opened with questions about participant demographics and professional experiences. Current and previous experiences with trauma charting were explored, were beliefs about the most important aspects of trauma charting and how a specific trauma form might change documentation and patient management. In the course of the interview, participants were given a paper copy of a proposed trauma form and time to review it, after which they were asked to provide feedback (Addditional file 2).

At all hospitals, interviews were held in a dedicated office or conference room away from the site of patient care. Written informed consent was gained from all participants prior to initiation of the interviews, and participants were not reimbursed for participating in the interview. Interviews lasted 30–45 min, conducted in the participant’s preferred language (Swahili), and were audio-recorded. All interviewers were all trained in qualitative methods and worked from the same interview guide.

### Data analysis

All interview transcripts were transcribed verbatim. The research team cross-checked the accuracy and completeness of translations against the original transcripts. Any gaps identified or clarifications needed were discussed and corrections made accordingly.

We used a hybrid thematic data analysis approach; this approach used both inductive and deductive reasoning (Fereday & Muir-Cochrane, 2006). We developed an initial codebook for data analysis, based on our study objectives. We then refined the codebook from the themes which emerged during the analysis. The first author developed the initial codebook and shared it with all authors. The codebook was discussed, further developed, and a final codebook was imported into qualitative data management and analysis software ATLAS.ti (Version 1.0.4,© ATLAS.ti, Berlin, Germany). The agreed codebook was tested by coding the first two interview transcripts by three authors. Their coding was almost similar and, hence, the codebook was not modified at this time. Transcripts were reviewed interactively for representative phrases, which were coded and grouped into themes. Categories and emerging themes were identified, using Braun and Clarke’s (Braun & Clarke, 2006) approach for thematic analysis (Braun & Clarke, 2006). Constant comparative methods and memos were used to develop codes, review interview questions, and make changes to develop the trauma form (Dicicco-Bloom & Crabtree, 2006). Inter-rater reliability was enhanced by using two reviewers who were each independently responsible for qualitative data analysis with equitable concordance of descriptive themes and sub-themes (Glaser et al, 1968).

### Ethical clearance

Ethical approval for this study was received from the University of Cape Town Human Research Ethics Committee and Muhimbili University of Health and Allied Sciences Institutional Review Board.

## Results

### Participants’ characteristics and experience with use of trauma form

A total of 33 healthcare providers were interviewed, with a median length of experience of 6 years (interquartile range (IQR): 2–7). Most (82%) were male. The job roles of the participants are shown in Table 1. Most (84.8%) of respondents had not used a standardised charting prior to this interview.

### Analytical themes and sub-themes

The findings of semi-structured interviews (SSI) revealed three themes:

1. Potential benefits of a trauma form,
2. Perspectives on the concept of a standardised trauma form, and
3. Concerns regarding successful and sustainable implementation.

**Fig. 1:**
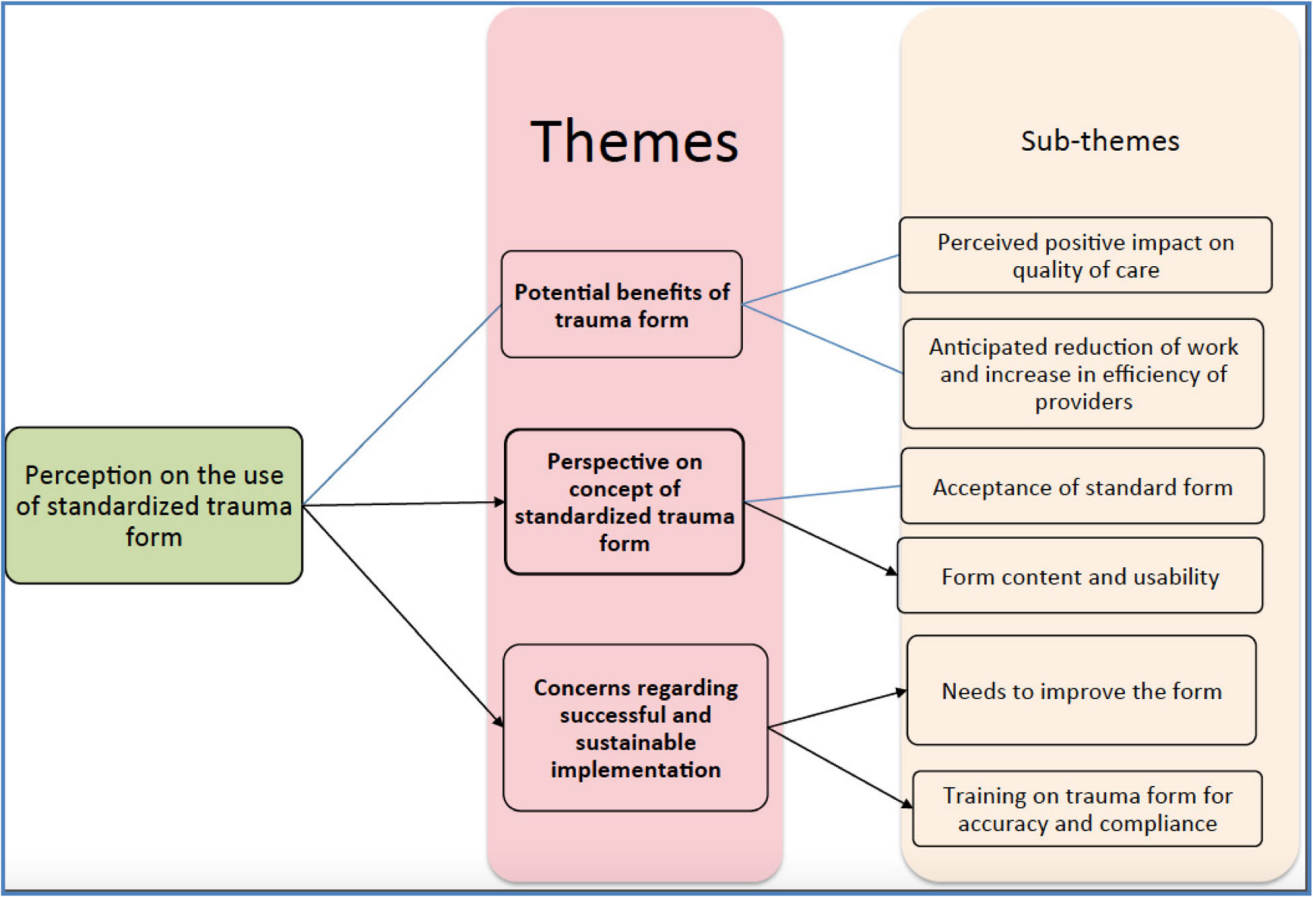
Analytical themes and sub-themes

These themes are supported by six sub-themes, as described below (Fig. 1).

### Theme one: potential benefits of trauma form

Several participants explained the potential benefits of having a standardised trauma form in their Emergency Unit (EU). These benefits ranged from individual facility benefit to collective national level matters.

#### Sub theme 1: perceived positive impact on quality of care

Many participants believed that the form would lead to improved quality of care within their hospitals. This perception emanated from the nature of design of the form, using mostly check boxes and hence serving as a prompt to the next most appropriate clinical intervention as well as reducing the amount of documentation required from the clinicians.

“*…*. I *will advocate to adopt this form as it is very useful because there are things I now realise we were omitting in evaluating trauma patients … ..for example, we do not document primary, secondary survey, we only write very important information … .but this is systematic …*” *[Participant no.25].*

One respondent noted how important it was to have the laboratory results listed on the form, as this would ensure accessibility in one platform and reduce the frequency of missing results.

“*… the fact that we are also documenting the findings on this form is very nice, because one of our major problem has been accessing the laboratory results, as there is frequent loss of documents …*” *[Participant no.30].*

#### Sub theme 2: anticipated reduction of work and increased efficiency of providers

Respondents perceived that having a standardised trauma form at the EU would reduce the clinical and administrative documentation workload that each facility is mandated to provide, such as manual recording in the HMIS register book.

“*..this form will make our work of filling MTUHA (HMIS) simpler as we will always have standard information when we need it …*” *[Participant no.8].*

Another respondent added that the use of the standard form will lead to more accuracy in recording due to reduced burden of recording in the MTUHA book.

“*… .yes indeed, I believe we shall be more accurate and efficiency as we shall have less burden of documentation, and MTUHA (HMIS) records can easily be pulled from this form even when the patient has already left.” [Participant- no.1].*

Furthermore, participants added that the availability of the form at the EUwill address the challenges of documenting legal cases, as mandated by the police force. They stated that the latter is essential as most of the trauma cases are treated as legal cases in Tanzania, and necessitate police permission to provide care, this permission being documented using a police form.

“*… .if the papers are filled properly and they are taken to registry, then when we need to file a PF3 [police legal form number 3] it will be much easier as this has been a challenge in the past..” [Participant no.23].*

### Theme two: perspective on the concept of standardised trauma form

This theme illustrates providers’ perceptions about the concept of using a standardised template trauma form for documentation.

#### Sub theme 3: acceptance of standard form

fRespondents commonly indicated a positive perception and acceptance of the use of standardised documentation across all sites. There was a general readiness to utilise the form and some participants advocated for immediate adoption of the form at their facility.

“*… yes, this is very usable form, and I think [name of hospital] should definitely adopt I; however, there are few areas I would change because as you know our resources are low …*” *[Participant no.9].*

They suggested that the standardised trauma documentation might serve as precursor to implementation of a Government HMIS (GOTHMIS) that is currently under development and implementation in some Regional Hospitals.

“*… this form is like a steppingstone to our new GOTHMIS plan [planned implementation of Government owned electronic medical record], hence I think we should move with it even though I think we may encounter some challenges during our initial stages …*” *[Participant no.13].*

#### Sub-theme 4: form content and usability

Respondents expressed widespread agreement on the form content. Most participants expressed satisfaction with variables and some noted that the form captured substantially more variables than were currently being documented.

“*…*. *this form is very comprehensive in all sections, as there are many variables that we are not normally documenting on regular basis, that I am seeing here for the first time but I think they are important …*” *[Participant no.6].*

Furthermore, the majority of participants perceived the form to be user friendly, in particular because of the use of checkboxes to reduce free text documentation. They believed that this would reduce their burden of documentation, especially given the large volume of patients and shortage of human personnel.

“*… this form looks user friendly because it has very little free text documentation, to the most it is check … check … .check [referring to checklist format] and you are done … I think will make my life very easy especially when we get overwhelmed by patients …*” *[Participant no.13].*

### Theme three: concerns regarding successful and sustainable implementation

Despite overwhelming support for and acceptance of the use of standardised documentation, some participants expressed concerns.

#### Sub-theme 5: needs to improve the form

While there was a strong agreement on the form’s setup and components, respondents expressed concern that the variables within the form might be too detailed for the facilities that do not provide advanced care for trauma patients. To reduce potential frustration, they proposed some modifications of the form that incorporate limited variables or components that may be available in their respective facilities.

“*… I see this as a good form, however there are several variables that are too detailed for patients we see at [name of hospital] since we refer most of severe injuries, such as those needing ORIF [open reduction and internal fixation], or TBIs [Traumatic Brain Injuries] … ..I think we should modify and reduce those [variables] to make it [ the form] more user friendly … …*” *[Participant no.1].*

Some interviewees highlighted concerns that prehospital details are not available if there is no prehospital care, and so the form needed to be adjusted to reflect this reality.

“*… .the section that ask details about patient before they get to our facility will be tricky because we don’t have ambulances except if the patient are referred to us from our health centres, but otherwise this information will be missing in most of our patients …*” *[Participant no.24].*

Furthermore, some of the participants expressed the need to create a mechanism within the form that would clearly show which variables were not completed as a result of lack of resources or capacity to provide required care; this was perceived as important for quality of care and, in event of medicolegal cases to give clarity on why the patient had not received particular care.

“*…*. I *think there are certain variables that are necessary to be included; for example, having the components related to lack of resources to care will help to show that we left the blank because was not performed due to lack of resources … this will help alleviate the burden of complaints that we often shoulder when there is an issue with the care of patient..”[Participant no.4].*

#### Sub theme 6: training on trauma form for accuracy and compliance

Participants noted a need for training of all providers in the use of the trauma form prior to its implementation, to ensure compliance and accuracy:

“*… one important issue will be training, as this form seems to have a lot of good information, but most of us [clinician] may document wrongly if we are not trained …*” *[Participant no.23].*

Another participant added:

“*… .awareness and training is key for both the doctors and nurses so as we remind each other during care, especially when there is mass casualty …*” *[Participant-4].*

In addition to the existing providers who need training to familiarise them with a new form, respondents highlighted the need to have regular and sustainable training beyond the implementation period because most of these facilities have doctors on brief rotations, who become primary care providers in the EU:

“*… … .you see we have interns here who rotate for 4 weeks, and they cover both night and day shifts, hence I think they will have to be trained regularly each time they come to our department for rotation … otherwise we will have only benefited those who are available during this period of implementation … ..”[Participant no.17].*

Some respondents expressed the need to have dedicated people who have additional training (similar to training of trainers) who will be the lead at each EU to ensure that the other providers receive continuous training, as the implementation is ongoing. This was idealised as a way to achieve regular and sustainable training that could last beyond the initial implementation phase. The participants said:

“*… ..I think the hospital should designate among us someone who will take one the role of trainer for all of us (champion), and he/she should receive additional training and become a leader for the registry … … since we have new staff regularly this model will help ensure there is ongoing training for each of new staff …*” *[Participant no.29].*

## Discussion

Results of these key informant interviews demonstrated support for adoption of a standardised trauma documentation form to be use as a clinical management guide to standardise care processes, reduce documentation burden, and improve data acquisition and reporting. It was clear that the majority of participants had not previously used standardised trauma documentation, or any standardised documentation; however, the majority had positive beliefs that a standardised template would bring benefits to patients, individual providers and the healthcare system at-large.

There was a wide acceptance and positive attitude towards use of standardized documentation across all the sites. Similar to observed impact of TR in most HICs (TARN, 2017) providers believe that successful implementation of standardised trauma documentation form at regional hospitals in Tanzania will have a wide reaching impact in care process as well as developing the first regional injury registry that is sustainable and can be replicated in other low income countries. Most of prior registries in Sub Saharan Africa have been largely limited, in part due to lack of engagement of providers in developing and implementing the form, as well as resource intensive nature of the design and modality of aggregating data (Nwomeh et al., 2006).

In this study, we found that the content of the proposed form overwhelmed some of the providers, who reported being unaware of some of variables in the form and called for their omission. In addition to the concern about provider knowledge, some participants highlighted resource challenges that may limit the capacity to completely fill in all of the variables according to the template. In contrast, other providers requested training to inform an understanding of unfamiliar variables, to improve compliance and accuracy of form completion. However, there was not agreement on whether to remove these variables, or leave them in, but with a means to record the lack of resources.

For example, most providers advised retaining the component on documentation of ultrasound finding, but adding a sub section of “not done” and “not indicated”. This would highlight a gap in care as well as reminder to clinicians of the importance of this assessment. None of the hospitals had a point of care ultrasound available, and this meant that the documentation of bedside ultrasound findings was not practical. Tailoring of the form to match the existing resources of the facility was perceived as one of the main factors that will determine sustainability and compliance towards successful implementation and utilisation.

Most providers expressed the need for training to support their understanding of the form content and utilisation, which might imply a limited understanding of the trauma care process. Our study did not assess the level of knowledge of respondents in providing trauma care; however, care gaps among trauma patients referred from regional to national hospitals are well-documented in-country (Lucumay et al., 2019). Also, in the present study, mistrust of care provision by junior and rotating clinicians further strengthened participants’ beliefs that robust training on the form will be necessary. Individual provider buy-in is central to implementation of new processes within health facilities (Karuza et al., 1995); a form such as this will be no exception. There was positive perception towards the use of the proposed trauma form, mostly owing to the view that the form, with its checklist nature, will improve care and reduce workload.

Implementation and use of standardised guideline and templates in LMICs is closely linked to the availability of the right human resources to provide optimal care (Kane et al., 2016). Contrary to our expectation, human resource shortages were perceived a strong rationale for implementation of a standardised form, as the form was expected to reduce the overall amount of time spent in documentation. The regular filing of HMIS to MoH, as well as reporting on police forms for all injured patients, was noted to be a daily burden for all healthcare providers. The majority of respondents also expressed the belief that the trauma form would enable the retrieval of the documentation of previous patients when necessary.

Implementation of registries in Sub Saharan Africa have been largely unsustainable, or limited within the research environment, mostly due to the nature of registry set up requiring continuous resources to sustain, as well as lack of provider engagement in supporting the implementation process (Bommakanti et al., 2018). Our study highlighted healthcare providers’ views on the utilisation of a standardised trauma form. These insights have provided crucial guidance towards the development and implementation process to ensure compliance and long-term sustainability. Finally, it is important that the development process of the trauma form takes into account the facility resources. This will ensure the form is fit for purpose across a healthcare system with variation in capacity to care for injured patients.

### Trustworthiness

Trustworthiness is attained in a qualitative study when the findings of such a study are worth believing (Graneheim & Lundman, 2004). In this study we adopted the four Guba criteria; credibility, dependability, transferability, and confirmability to enhance the trustworthiness (Shenton, 2004). The credibility of the findings of this study was enhanced through the triangulation of informants with experiences and rich information on the study questions. In order to enhance the credibility and dependability of this study, we used the triangulation of informants, study settings, and researchers. Data were collected using a semi-structured interview guide that allowed for probing. In order to confirm that the findings reflected informants’ perspectives rather than the researchers’ understanding of the question under study, themes we inductively generated using thematic analysis approach and presented with the support of sub-themes and quotes. The transferability of the findings of this study is enhanced through the description of the study setting, context, data collection process, and analysis.

### Limitations

As for other qualitative studies, the generalisability of this study is likely limited by the small sample size of healthcare providers and number of sites. However, the thick description of the study context provides room for the transferability and thus applicability of these findings in other similar contexts. Interviews were conducted in Kiswahili, transcribed verbatim, and translated into English language for analysis, which might have introduced some errors. To counteract this effect, two authors reviewed the translated scripts.

## Conclusion

Findings of this study revealed wide healthcare provider acceptance of moving towards standardised clinical documentation for trauma patients. Successful implementation likely depends on the perceived benefits of using a trauma form as a tool to guide clinical management, standardise care and standardise data reporting; however, it will be important moving forward to factor concerns brought up in this study. Potential barriers to successful and sustainable implementation of the form, including the need for training and tailoring of form to match existing resources and knowledge of providers, must be considered.

## Supplementary information

**Additional file 1.**

**Additional file 2.**

## Data Availability

The datasets used and/or analysed during the current study are presented as additional supporting files in this manuscript.
