# Supplementary material for: Perceptions of health providers towards the use of standardised trauma form in managing trauma patients: a qualitative study from Tanzania
Source: Inj Epidemiol. 2020 May 1;7:15. doi: 10.1186/s40621-020-00244-3 (PMC7193390; doi:10.1186/s40621-020-00244-3)
Supplement: Supplementary file 1 — Additional file 1. [file 40621_2020_244_MOESM1_ESM.pdf]

REGIONAL HOSPITAL TRAUMA FORM

Hospital Registration Number:	Date: DD/MM/YY Time of Arrival: : AM/PM
Patient Name (Surname, First):<br>Occupation:	Arrival Mode: □Walk □Non-motorized vehicle □Private vehicle<br>□Motorized 2- or 3-wheeler □Taxi □Public transport □Police<br>□Ambulance □ Aeromedical □Unknown □Other: ___________
Date of Birth: DD/MM/YY Age: ________	
Sex: M / F Weight: kg INF / CH / AD	# prior facilities: ____ Referred from: ____________________
Patient Residence (at least City and Sub-district):<br>Sub-district where injury occurred:	□Ambulatory Non Ambulatory: □ Acute □ Chronic<br>Contact Person:<br>Phone: Relation:

CHIEF COMPLAINT:

Triage Category: ____________ □ **Mass Casualty**

INITIAL VS: **Time:** : AM/PM

□ **Dead on arrival**

Temp: _____ **BP:** _____ **/**_____ **HR:**_____ **RR:**_____ **SpO_2:**_____ **% on**___ L
Pain score (on a scale of 1-10, see Reference Card for details): ________

FIRST PROVIDER EXAM:
Date: DD/MM/YY Time: : AM/PM

PRIMARY SURVEY (see Reference Card for normal findings, only mark NML if all key elements are normal)**:**

**A**irway<br>□ NML	□ Angioedema □ Stridor □ Voice changes<br>□ Oral/Airway burns<br>**Obstructed by**: □ Tongue □ Blood □ Secretions<br>□ Vomit □ Foreign body	**Airway Manipulation:** □ Repositioning □ Suction<br>**Airway:** □ OPA □ NPA □ LMA □ BVM □ ETT<br>**Cervical collar:** □None needed □Placed before arrival □Placed in EU<br>(none needed = not altered, no pain or TTP, no distracting injury)	
**B**reathing<br>□ NML	**Spontaneous Respiration:** □ Yes □ No<br>**Chest Rise:** □Shallow □Retractions □Paradoxical<br>**Trachea:** □ Midline □Deviated to □L □R<br>**Breath Sounds:**<br>Abnormal: □ L___________ □ R____________	**Oxygen:** _____ L<br>□NC □Mask □NRB □BVM<br>□CPAP/BIPAP □Ventilator	**Chest needle / tube** (circle):<br>□ L – Size: ________<br>Depth: ________ cm<br>□ R – Size: ________<br>Depth: ________ cm
**C**irculation<br>□ NML	**Skin:** □ Warm □ Dry<br>□ Pale □ Cyanotic □ Moist □ Cool<br>**Capillary refill:** □ <2 sec □ ≥2 sec<br>**Pulses:** □ Weak □ Asymmetric______<br>**JVD:** □ Yes □No	□ Bleeding controlled (bandage, tourniquet, direct pressure)<br>**Access:** □IV: Loc ___________ Size _____<br>□CVL: Loc ______ Size _____ □IO: Loc _______ Size _____<br>□**IVF:**__________mLs □NS □LR □Other___________<br>□Blood ordered □Pelvic binder placed	
**D**isability<br>□ NML<br>**E**xposure<br>□ NML	**Blood glucose**: ____________ □Glucose<br>**Responsiveness:** □ A □ V □ P □ U □Naloxone<br>**GCS**:______ (E______ V______M______)<br>**Moves Extremities:** □ LUE □RUE □LLE □RLE<br>**Pupils:** L____mm→____mm R____mm→____mm<br>□ Exposed completely	□ Not Indicated<br>**F**AST<br>□ NML	**Peritoneum:** □ Negative □ Indeterminate<br>□ Free Fluid: ________________________<br>**Chest:** □ Negative □ Indeterminate<br>□ Pneumothorax (R/L): __________________<br>□ Pleural fluid (R/L): ___________________<br>□ Pericardial effusion

MEDICAL HISTORY

Medications:

Allergies:

Past Medical: □ HTN □ Diabetes □ COPD □ Psychiatric □ Renal Disease
Other:

Pregnant: □ Yes □ No
Vaccinations up to date? □Yes □No ______________
Substance Use: □Tobacco □Alcohol □ Drugs □ IV Drugs
Last Menstrual Cycle:_______□ N/A **G**____**P**____ □ N/A
Safe at home? ___________________________

Past Surgeries (type & date)**:**

HISTORY OF PRESENT ILLNESS

Date of Injury: DD/MM/YY Time: : AM/PM

Enter exact term from Reference Card for the following:
Place of injury: ___________________________________

Prehospital care: ____
Patient's activity of time of injury: ____________

Mechanism of injury:
□Road traffic incident: □ Driver □ Passenger □ Pedestrian
□ Airbag □ Seat belt □ Other vehicle restraint □ Helmet
□ Extricated Vehicle involved: ______________________
□ Ejected Crashed with: ________________________
□Fall from: ____________ □Hit by falling object: ____________
□Stab/Cut □Gunshot □Sexual Assault
□Other blunt force trauma (struck/hit): ___________________________
□Suffocation, choking, hanging
□Drowning: ____________________________ Flotation device: Y / N
□Burn caused by: _____________________________________
□Poisoning/Toxic Exposure:_____________________________________
□Unknown □Other: ___________________________

Intent: □ Unintentional or accidental □ Intentional: □Self harm □Assault
□ Legal process, political unrest or war □ Unknown
Assaulted by (see Reference Card): ____________________________

Hours since last meal: ______________ HR

Substance use within 6 hours of injury:
□ Unknown □ None □ Reported □ Evidence (positive test or clinical findings)
□ Alcohol □ Other Substance (if known): ________________________

Details of Incident:
□ LOSS OF CONSCIOUSNESS: <5 min/ 5-29 min/ 30-24 hr/ > 24 hr
□ TRAUMA: □ Head □ Neck □ Chest

PHYSICAL EXAM: (See Reference Card for normal findings. Do NOT mark NML unless all key elements are normal.)

□NML	**General**	
□NML	**HEENT**	
□NML	**Neuro**	
□NML	**Neck**	
□NML	**Pulm/Chest**	
□NML	**Cardiac**	
□NML	**Abdominal**	
□NML	**Pelvis**	
□NML	**GU/Rectal**	
□NML	**Back**	
□NML	**MSK/Skin**	

Detail area of injury:

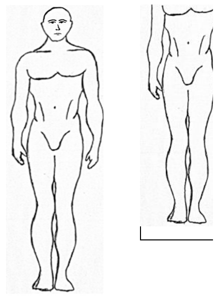

LAB RESULTS:

UPT: □ Positive □Negative
Hgb: ______________ □ Result pending
Blood type: ______________
Other:

IMAGING RESULTS:

□ Pneumothorax □ Pleural Fluid □ Rib Fracture □ Pulmonary Opacity
□ C-spine fracture □ Extremity Fracture □ Pelvic Fracture
□ Wide mediastinum
□ Other:

ADDITIONAL INTERVENTIONS:

Fluids and Medications Given (include time)
□ IVF:__________mLs □NS □LR □Other
□ Blood products (specify number of units given):
Whole Blood_____ PRBC_____
FFP_____ Platelets____
□ Opioid Analgesia:______________________
□ Other Analgesia:______________________
□ Sedation and Paralytics:________________
□ Antibiotics:__________________________
□ Tetanus: ___________________________
□ Other:_____________________________

Procedures (include time and outcome)
□ Cricothyroidotomy: Open / Needle_________________________________
□ Intubation: ____
□ Chest Tube: ____
□ Pericardiocentesis: ____
□ Open Thoracotomy: ____
□ Splinting: ____
□ Fracture Reduction/Pelvic Stabilisation: ____________________________
□ Foreign Body Removal:____
□ Simple / Complex Laceration Repair: ______________________________
□ Other: ____

ASSESSMENT (include summary and differential) **AND PLAN** (imaging, meds/interventions, consults, etc):

Consultants (time called, time arrived, recommendations)**:**

REASSESSMENT at ____:____ AM/PM.
Temp HR BP / RR SpO_2: % on L

Condition: □ Same
□ Changed: ____________________

DISPOSITION Checklist completed: □ Y □ N ED departure (date & time): DD/MM/YY : AM/PM

Diagnoses/Impressions (list all):

Number of serious injuries (circle): 0 1 ≥2

□ **Admit to:** □ Ward __________ □ ICU □ OT
□ **Discharge** Plan discussed with patient?: □ Yes □ No
□ **Transferred to**: ______________________________ Accepting Provider: ________________________________
□ **Died of** (specify cause-NOT cardiopulmonary arrest): ____
□ **Left without being seen** □ **Left without complete treatment**

Provider	Role	Signature and Date
