# Supplementary material for: Perceptions of health providers towards the use of standardised trauma form in managing trauma patients: a qualitative study from Tanzania
Source: Inj Epidemiol. 2020 May 1;7:15. doi: 10.1186/s40621-020-00244-3 (PMC7193390; doi:10.1186/s40621-020-00244-3)
Supplement: Supplementary file 2 — Additional file 2. [file 40621_2020_244_MOESM2_ESM.doc]

## Appendix 2: Semi-structured interview guide

**Part I:**

| - **Tell him/her**: Thank you for taking time out from your busy schedule to do this   interview.   - **Inform him/her**: That his/her identity will be kept confidential throughout the   investigation.   - **Inform him/her**: Initial analysis of the capture rate for each of the variables of the WHO minimum data set for injury among trauma patients at *[Hospital]* is not consistently well done. Our aim is to improve the capture rate of all variables within the WHO minimum dataset for injuries through development of a standardized documentation chart. - **Introduction**: As a key personnel of the emergency unit here at [*Hospital*] I would value your personal input in the design and piloting of a trauma chart for the [*hospital*] emergency providers. This document could be utilized to document trauma patients during care and collect information on injury coming through your facility. The interview should take approximately 45 minutes, we will ask a few questions and then show you a draft of the trauma chart for your suggestions and comments. All your responses will remain anonymous, and you are free to terminate the interview at anytime. We will record this interview for transcription purposes. - **Let him/her**: Read and sign the informed consent form if they agree to do. - Remember to thank him/her after the interview. |
| --- |

1. **Personal details and professional experience:**
2. What is your position at the emergency unit?
3. How long have you worked as a provider, post graduation?
4. How many years of experience do you at the emergency department?
5. Where did you train?
6. Have you worked in other hospitals other than *[Hospital]?*
7. **Past and current trauma charting:**
8. Have you ever used a trauma form? (**Probe:** Why or why not?)
9. Are you currently using a form? (**Probe:** Why or why not?)
10. If not, how do you currently chart trauma patients?
11. When do you normally do your charting (during treatment, patient still in emergency unit, after stabilized)?
12. **After reviewing the proposed Form**:
13. How usable is this form? Would you and other providers at [*Hospital*] use it?
14. Does it capture the needed information? (**Probe:** If not, what is missing?)
15. What makes this form useful?
16. What makes this form difficult? (**Probe:** what would be the challenges with using the form)?
17. Is there anything you would add or subtract from the proposed draft form?
18. **After piloting testing the form**:
19. How usable is this form?
20. Does the form simplify your charting of the trauma patients (**Probe:** why/why not)
21. What makes this form difficult to use? (**Probe**: why?)
22. Which aspects of the form would you like to be changed (**Probe**: why?)

**Part II**
